# Total Pancreatectomy for Malignant Intraductal Papillary Mucinous Neoplasm (IPMN) Complicated by Gastropancreatic Fistulae

**DOI:** 10.1155/2020/8547526

**Published:** 2020-03-24

**Authors:** Oshan Basnayake, Pradeep Wijerathne, Umesh Jayarajah, Nilesh Fernandopulle, Sivasuriya Sivaganesh

**Affiliations:** ^1^Professorial Surgical Unit, National Hospital of Sri Lanka, Colombo, Sri Lanka; ^2^Department of Surgery, Faculty of Medicine, University of Colombo, Colombo, Sri Lanka

## Abstract

**Background:**

Intraductal papillary mucinous neoplasms (IPMN) of the pancreas complicated by fistula formation to adjacent organs are an uncommon phenomenon. We present an IPMN of the pancreas with malignant transformation and multiple fistulae to the stomach and duodenum. *Case Presentation*. A 50-year-old female was referred for investigation of recent epigastric pain and a past history of recurrent pancreatitis. Imaging with computed tomography showed a gross dilatation of the entire pancreatic duct with a heterogeneous enhancement of the periductal parenchyma. A passage of oral contrast was noted from the greater curvature and pylorus of the stomach into the dilated duct suggestive of fistulae formation. Gastroduodenoscopy demonstrated these fistulae in the stomach and the proximal duodenum and an exophytic growth at the ampulla obliterating the view of ampullary opening. Endosonography- (EUS-) guided fine-needle aspiration cytology (FNAC) showed cells with high-grade atypia. A total pancreatectomy, distal gastrectomy, and splenectomy were performed, and recovery was uneventful. Histology revealed a ductal adenocarcinoma arising from an intestinal type intraductal papillary mucinous neoplasm with high-grade dysplasia. A year and a half after surgery, she is healthy with good glycaemic control and nutritional status.

**Conclusion:**

This case highlights the importance investigating patients for the aetiology in recurrent acute pancreatitis and their follow-up. Awareness of cystic pancreatic neoplasms including IPMN is important to avoid misdiagnosis or delayed diagnosis. Referral of these patients to centres with facilities for multidisciplinary input and specialised management is strongly recommended.

## 1. Introduction

Intraductal papillary mucinous neoplasms (IPMN) of the pancreas are cystic neoplasms composed mainly of mucous-secreting columnar cells with variable cellular atypia [1]. The usual age of presentation is between the fifth and seventh decades with equal distribution among the sexes and a prevalence of 2-45% in general population [1]. Depending on the duct involvement, these tumours are classified as main duct type, branch duct type, or mixed type. Histologically, these tumours are classified into intestinal, pancreaticobiliary, oncocytic, and gastric types. The risk of invasive carcinoma in high-risk IPMN was 12% over a mean follow-up period of 60 months [2]. Fistula formation to adjacent organs in IPMN with invasive carcinoma is an uncommon phenomenon. A retrospective study using computed tomography (CT) and magnetic resonance imaging (MRI) of 423 patients with IPMN showed fistulae involving the duodenum, stomach, common bile duct, and colon in 1.9% (*n* = 8). Furthermore, fistulae appeared to predominate in malignant main duct IPMN [3]. We present a case of a colloid type ductal adenocarcinoma of intestinal variety occurring in the background of IPMN with multiple fistulae to the greater curvature of the stomach, pylorus, and duodenum.

## 2. Case Presentation

**Figure 1:**
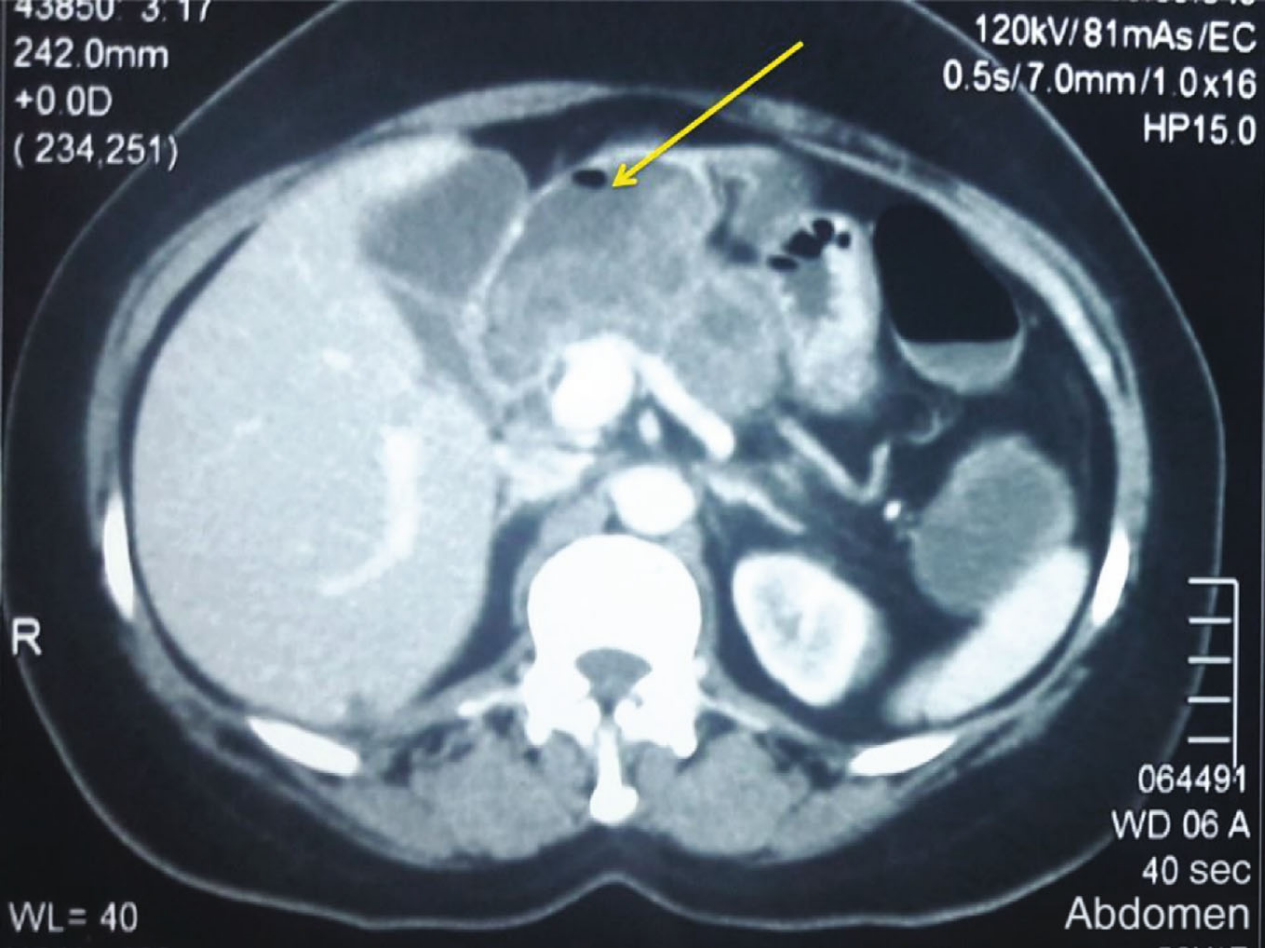
The yellow arrow shows the cystic neoplasm of the pancreas with air inside.

**Figure 2:**
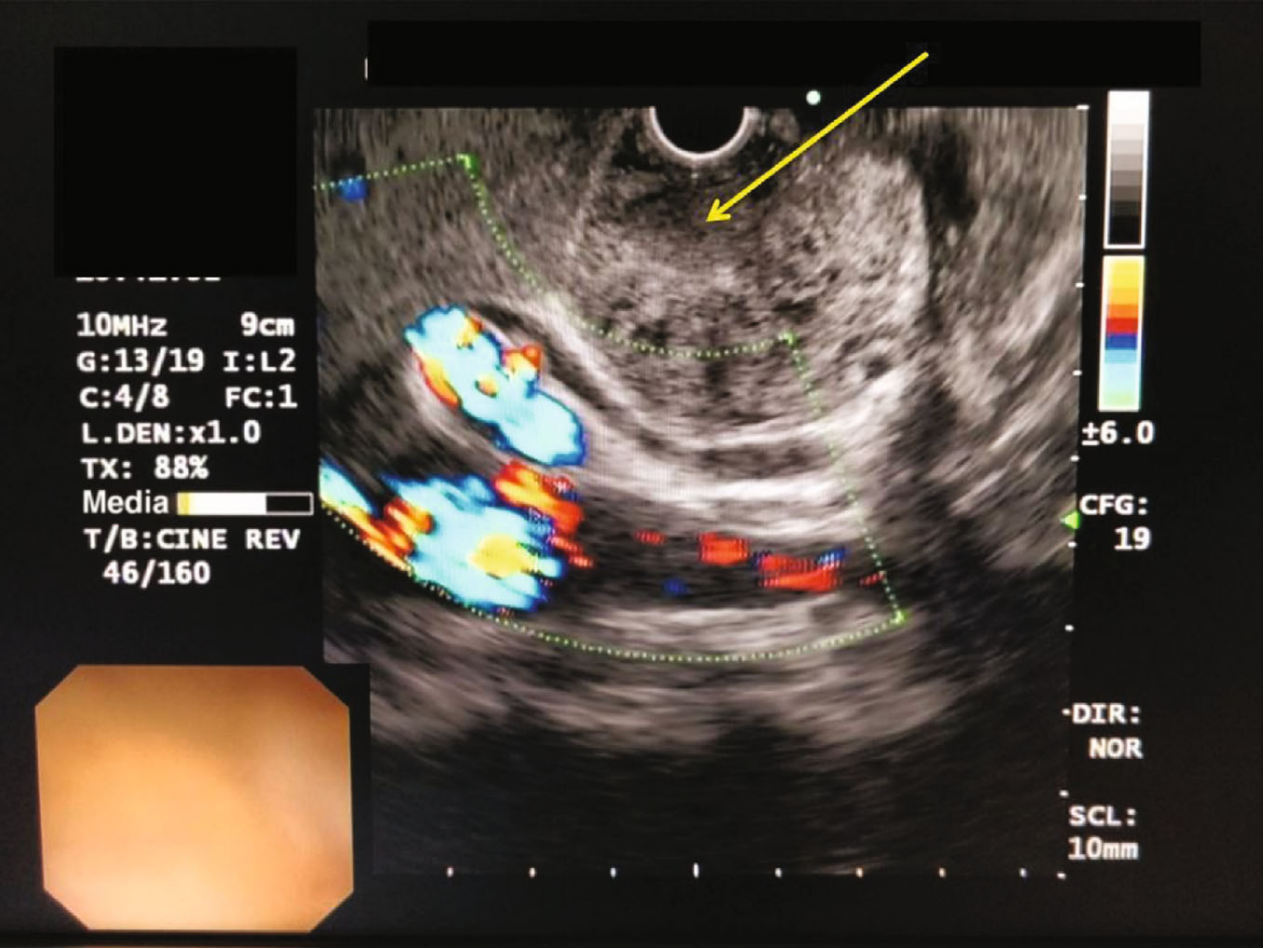
Endoscopic ultrasound (the yellow arrow shows a mixed echogenic collection in relation to pancreatic head and body).

A 50-year-old female was referred for investigation of recurrent epigastric pain. She was otherwise well with no constitutional symptoms, anorexia, or weight loss and was normoglycaemic. She had a past history of recurrent pancreatitis with elevated amylase levels. The liver enzymes and abdominal ultrasonographic studies were normal. She has been asymptomatic for the preceding 3 years. A contrast-enhanced CT scan which was performed 3 years ago had been unremarkable except for evidence of acute pancreatitis and a 6 mm main pancreatic duct dilatation. The contrast-enhanced CT showed a gross dilatation of the entire pancreatic duct (diameter = 23 mm) with a heterogeneous enhancement of the periductal parenchyma of the whole gland with an irregular outline. A passage of oral contrast was noted from the greater curvature and pylorus of the stomach into the dilated duct suggestive of fistulae formation (Figure 1). Gastroduodenoscopy demonstrated these fistulae in the stomach and also the proximal duodenum and exophytic growth at the ampulla obliterating the view of ampullary opening. Endosonography (EUS) revealed a mixed echogenic collection in relation to pancreatic head and body (Figure 2), and the EUS-guided fine-needle aspiration cytology (FNAC) showed cells with high-grade atypia. The volume of aspirate was inadequate to perform carcinoembryonic antigen (CEA) and amylase assays. Her preoperative CA 19.9 level was 21 U/mL, and faecal elastase was not performed.

**Figure 3:**
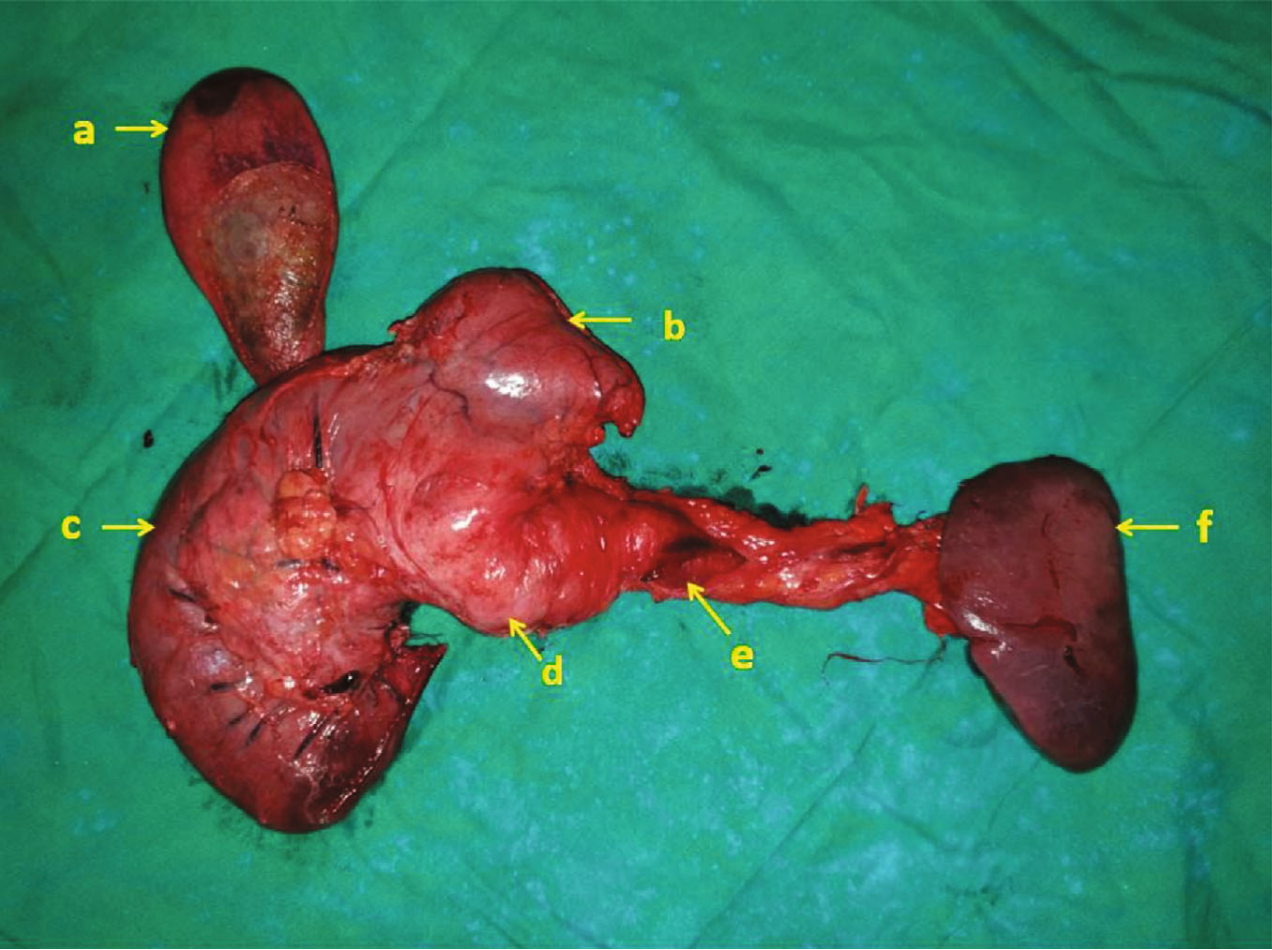
Postoperative specimen. a: gall bladder; b: distal stomach; c: duodenum; d: pancreas; e: fistulating part from the proximal stomach; f: spleen.

She underwent a total pancreatectomy, distal gastrectomy, and splenectomy. The fistula in the proximal stomach was removed with a cuff of posterior gastric wall using a linear stapler (Figure 3). The postoperative recovery was uneventful, and glycaemic control was achieved with a basal-bolus insulin regime. A histological analysis revealed a ductal adenocarcinoma (colloid type) arising from an intestinal type intraductal papillary mucinous neoplasm with high-grade dysplasia and pathological stage of pT3N0Mx. She was prescribed insulin and pancreatic enzyme supplementation, received postsplenectomy vaccination, and is on penicillin prophylaxis. She received FOLFIRINOX (folinic acid, fluorouracil, irinotecan, and oxaliplatin) 2 weekly for 12 cycles as adjuvant chemotherapy. At 18 months after surgery, she is disease-free with good glycaemic control and nutritional status.

## 3. Discussion and Conclusion

Most patients with IPMN are asymptomatic and are often diagnosed incidentally. Others present with symptoms of pancreatitis due to ductal obstruction by mucin or nonspecific abdominal symptoms. Late diagnosis is not uncommon due to the insidious onset and slow progression of the disease and also lack of awareness among clinicians who do not infrequently mistake abnormalities on imaging to be due to pancreatitis. Proper evaluation of patients with cystic lesions in pancreas should be considered especially in patients with late-onset pancreatic type pain. Evaluation includes cross-sectional imaging, biochemical assay, and cytology of aspirate obtained at endosonography. Contrast CT has an accuracy of 40-81% [4, 5] while MRI has a higher accuracy of 40-95% but with equivalent specificity [6, 7]. Endosonographic differentiation of benign and malignant lesions is subject to expertise but has the advantage of obtaining samples for cytology and biochemistry [8]. The side-viewing endoscopic appearance of bulging ampulla extruding thick mucus is a characteristic finding of IPMN [8].

IPMN complicated by fistula formation to the adjacent viscera has been reported. The viscera involved include the duodenum, stomach, and bile duct, and 39% were multiple [9]. Direct tumour invasion and mechanical pressure in the setting of noninvasive tumours are proposed mechanisms of pathogenesis of fistula formation [9].

The risk of invasive carcinoma in high-risk IPMN was 12% over a mean follow-up period of 60 months [2]. The established risk factors for malignancy include the presence of jaundice, enhancing mural nodules, solid components, and a main pancreatic duct (MPD) diameter of >10 mm. Because of the time-dependent risk of progression to a malignancy, surgery is indicated in high-risk patients [10]. A duct diameter of >10 mm is an absolute indication for surgical resection of MPD IPMN while a diameter of 5-9.9 mm is considered a relative indication [11]. Options for surgical resection include pancreaticoduodenectomy with intraoperative frozen section assessment of the resection margin and total pancreatectomy. Data on the optimal type of surgical resection for main duct IPMN are conflicting [12]. Patients who undergo pancreaticoduodenectomy require close surveillance for early detection of malignant recurrence followed by completion pancreatectomy when suspected. Relative preservation of pancreatic function is achieved with pancreaticoduodenectomy but at the cost of surveillance. Total pancreatectomy is recommended in patients with involvement of entire MPD because of the elevated risk of high-grade dysplasia and cancer [10]. Loss requiring replacement therapy of both endocrine and exocrine functions is the downside of total pancreatectomy.

Irrespective of lymph node status, adjuvant systemic chemotherapy is often recommended because of the associated aggressive behaviour of these malignancies [13].

## 4. Conclusion

This case highlights the importance of investigating those with acute relapsing pancreatitis for aetiology and their follow-up. Awareness of cystic pancreatic neoplasms including IPMN is crucial in clinical practice to avoid misdiagnosis or delayed diagnosis. In the management of these patients, a multidisciplinary approach is strongly recommended. This is the first reported case of successful surgical management of a malignant main duct IPMN by total pancreatectomy in Sri Lanka.

## Abbreviations

IPMN: Intraductal papillary mucinous neoplasm
EUS: Endosonography
CT: Computed tomography
MRI: Magnetic resonance imaging
CEA: Carcinoembryonic antigen.
